# Ocular Manifestations of a Novel Proximal 19p13.3 Microdeletion

**DOI:** 10.1155/2018/2492437

**Published:** 2018-04-30

**Authors:** L. Swan, D. Coman

**Affiliations:** ^1^Department of Paediatrics, The Wesley Hospital, Brisbane, QLD, Australia; ^2^Discipline of Paediatrics, UnitingCare Clinical School, Brisbane, QLD, Australia; ^3^School of Medicine, Griffith University, Gold Coast, QLD, Australia; ^4^School of Medicine, The University of Queensland, Brisbane, QLD, Australia; ^5^Neuroscience Department, Lady Cilento Children's Hospital, Brisbane, QLD, Australia

## Abstract

Microdeletions at 19p13.3 are rarely reported in the medical literature with significant phenotypic variability. Among the reported cases, common clinical manifestations have included developmental delay, facial dysmorphism, and hypotonia. Herein we described a child with a de novo 19p13.3 microdeletion, proximal to the reported cases of 19p13.3 microdeletion/duplication, with ocular manifestations of bilateral ocular colobomata complicated with microphthalmos and cataract, associated with short stature. This case highlights the phenotypic heterogeneity of deletions in the 19p13.3 region.

## 1. Introduction

Chromosome 19 has been identified as having the highest gene density within the human chromosomes, with large gene families of evolutionary significance [1]. Deletions and duplications within the terminal band, 19p13.3, are sparsely reported in the medical literature with significant phenotypic variability. Most patients described within the literature have developmental delay, macrocephaly, and hypotonia with dysmorphic features [2–10]. Risheg et al. described two cases where one case had dysmorphic features including downslanting palpebral fissures, prominent auricular root, cupped ears, and mouth abnormalities [7]. The second case also included ear anomalies, notably a unilateral preauricular skin tag, and helix anomalies [7].

It would also appear that there exist common dysmorphic features within 19p13.3 microdeletions, with various ear abnormalities being the most common association. Deletions at 19p13.3 have been associated with ophthalmologic issues such as amblyopia, myopia, and strabismus as well as congenital cardiac issues [2–4, 6–9]. A comprehensive summary of previously reported phenotypic features is provided in Table 1.

19p13.3 microdeletion/duplication syndrome has been previously described within the literature; however, the shortest region of overlap (SRO) is approximately 150 kb and includes one microRNA (SNORD37) and four genes (PIAS4, ZBTB7A, MAPK2, and partially EEF2). Herein we report a case of a child with a novel deletion, proximal to the SRO, resulting in additional novel ocular structural anomalies.

## 2. Case Report

### 2.1. Clinical Case

We report a 6-year-old female born to nonconsanguineous parents. Antenatal history is unremarkable, with mother reporting a normal pregnancy and delivery via an elective caesarean section at 38 weeks. Birthweight was 2800 grams (10th%), head circumference was 32 cm (25th%), and length was 45 cm (3rd%) with Apgar scores of 9 at one minute and 9 at 10 minutes. The child was slow to reach early developmental milestones, approximately 6 months behind age appropriate milestone attainment, especially in the domains in gross motor and speech and language.

At age 5, she was assessed by an ophthalmologist and diagnosed with bilateral chorioretinal coloboma, left iris coloboma, left cataract, left sensory exotropia, and left microphthalmos. She was found to have normal vision in her right eye, but left eye was legally blind with only light perception intact. Exploration of her eyes under anaesthesia found left inferior chorioretinal coloboma involving the optic nerve but not the macular with vitreous detachment. It also found a right inferior chorioretinal coloboma not involving the macula. She has an area of dermal hypoplasia in the region of her left occiput, is nondysmorphic, and has an otherwise normal systems review to clinical examination.

She continued to maintain growth trajectories with the predominate feature of short stature (below 1st centile). Her growth parameters at five years of age were weight 12 kg (below 1st centile), head circumference 44 cm (25th%), and height 92 cm (below 1st centile) and currently at 6 years of age were height 99.5 cm (below 1st centile), weight of 14 kg (below 1st centile), and head circumference 44 cm (25th%).

A brain magnetic resonance imaging scan was normal with no signs of a neuronal migration defect. Transferrin isoforms were performed as a screening test for the congential disorders of glycosylation which were also normal. Baseline renal ultrasounds and cardiac echocardiograms were also normal.

## 3. Methods

Genome analysis was completed by Illumina Whole Genome Infinium CytoSNP 850K Array V1.1 with results analysed with BlueFuse Multi version 4.3, for the patient and subsequently her phenotypically normal parents.

## 4. Results

A de novo 533 kb deletion arr[GRCh37] 19p13.3(4399876_4933228) x1 was identified. This is proximal to (and does not encompass) the region reported in association with 19p13.3 microdeletion/microduplication syndrome [PMID: 25853300 and 23610052], containing 21 known genes between RefSeq genes including* CHAF1A* (chromatin assembly factor 1, subunit A; OMIM 601246),* UBXN6* (UBX domain protein 6; OMIM 611946),* PLIN4* (perilipin 4; OMIM 613247),* PLIN 5* (perilipin 5; OMIM 613248),* LRG1* (leucine-rich alpha-2-glycoprotein; OMIM 611289),* SEMA6B* (semaphoring 6B; OMIM 608873),* SF20* (stroma-derived growth factor 20; OMIM 606746),* TRNAG3* (transfer RNA glycine 3; OMIM 615303),* TRNAV32 *(transfer RNA valine 32; OMIM 615304),* FEM1A* (Fem 1 homolog A; OMIM 613538),* TICAM1 *(TIR domain containing adaptor molecule 1; OMIM 607601),* PLIN3* (Perilipin 3; OMIM 602702),* UHRF1* (ubiquitin-like protein containing PHD and ring finger domains 1; OMIM 607990), and* KDM4B* (lysine-specific demethylase 4B; OMIM 609765). Of these,* TICAM1 *is the only OMIM listed disease causing gene.

## 5. Discussion

Ocular colobomata typically is the result of embryological defective closure of the fetal or choroidal fissure at the anterior portion of the optic stalk during the 5th to 7th week [10]. The fetal fissure is located in the inferonasal quadrant, with this quadrant extending from the iris through to the optic nerve [10]. Should defective closure occur, any part of this quadrant and any structure contained within it may be affected, including the optic nerve [10]. A typical coloboma is located in the inferonasal quadrant, with an atypical coloboma existing anywhere outside of this location [10].

Colobomata is a genetically heterogenous ocular anomaly. Alterations in dosage-sensitive transcription factor genes (such as* SOX2 *(OMIM 184429)*, OTX2* (OMIM 600037), and* PAX6 *(OMIM 607181)) and retinoic acid metabolism genes (such as* STRA6 *(OMIM 610745)*, ALDH1A3 *(OMIM 600463), and* RARB *(OMIM 180220)) [13]. Ocular colobomata is a common feature and part of the CHARGE syndrome (OMIM 214880) phenotype. CHARGE syndrome, caused by a mutation in* CHD7* (OMIM 608892), displays coloboma of the retina and choroid [14]. Rainger et al. described cases of bilateral coloboma caused by a defect in* Mab21l2* (OMIM 604357) identified on whole exome sequencing [15]. Treacher Collins syndrome (OMIM 154500) has also been linked to ocular coloboma [16]. Other associations reported within the literature include oral-facial-digital syndrome X (OMIM 311200), 5q22.3q23.3 deletion, Baraitser-Winter syndrome (OMIM 243310), Walker-Warburg syndrome (OMIM 236670), Joubert syndrome II (OMIM 608091), Noonan syndrome (OMIM 163950), Rubinstein-Taybi syndrome (OMIM 180849), Meckel-Gruber syndrome (OMIM 249000), Goltz syndrome (OMIM 305600), Aicardi syndrome (OMIM 304050), and Lenz syndrome (OMIM 309800) [13, 16, 17]. Chromosomal aberrations previously described within the literature linked to ocular coloboma are so far limited to chromosomes 2, 4, 7, 11, 8, 16, and 22 [16].

In addition to colobomata, the patient described here also possessed a complex microphthalmia complicated by cataract. This defect has not been previously described by the literature in association with a deletion at 19p13.3. Hurgoiu and Suciu (1984) described a case with primitive vitreous body and punched out lesions within the retina, but with no further molecular characterization other than 19p13-pter [18]. Palumbo et al. described a case with exotropia [3], whereas Siggberg et al. described a case with severe amblyopia and hyperopia [6]. Cases described by Risheg et al. and Kuroda et al. possessed eye abnormalities but the extent of the defects was myopia and ptosis [7, 11]. Furthermore, Nevado et al. described multiple cases of microdeletion involving phenotypic eye anomalies, including amblyopia, astigmatism, and myopia [2].

Our patients 19p13.3 microdeletion (4,399,876_4,933,228) is proximal to a region reported in association with “19p13.3 microdeletion/microduplication syndrome” [2]. This indicates that this is a previously undescribed deletion within the 19p13.3 region. A search of the DECIPHER database (https://decipher.sanger.ac.uk/) did not find any corresponding previously reported deletion within the exact region; however, several overlapping deletions with different phenotypes have been described (see Table 1). Each of these overlapping deletions has failed to describe an ocular anomaly, like the one seen in our patient.

Of the genes that span this region,* KDM4B* has been found in murine models to be active during embryogenesis, specifically within the visual system [19]. It could be theorised, therefore, that the deletion of a portion of this gene could profoundly affect the embryological development of the eye seen in this case study [19]. Additionally,* UHRF1* has been found, in murine models, to be localised within the visual system in early embryological development; however, human research is lacking [20]. This may also theoretically cause an additive effect on the deletion outlined in this case.* LRG1* has also been demonstrated to have a role in retinal development, with mutation encompassing this gene theoretically implicated in abnormal retinal development [21]. Another gene mapped to this area of 19p13.3 is also* CHAF1A*, which may have some functional impact and similarities to* CHD7*, the causative gene of CHARGE syndrome [14]. It is also hypothesised that LRG1 plays an important role in retinal angiogenesis [22]. Other genes mapped to this area have not been shown to have a role in oculogenesis either in murine models or in human models [23].

In summary, this case study adds further information about chromosome 19p13.3 deletions and the significant phenotypic characteristics of those affected. Further research needs to be completed on the causative genes for further elucidation of the cause of the ocular anomalies within this child.

## Figures and Tables

**Table 1:** Summary of phenotypic features.

	Our Patient	Case 1 [9]	Case 2 [9]	Case 3 [9]	Case 4 [9]	Case 5 [9]	Case 6 [9]	Case 7 [9]	Case 8 [9]	Case 9 [11]	Case 10 [7]	Case 11 [7]	Case 12 [7]	Case 13 [4]	Case 14 [5]	Case 15 [12]	Case 16 [6]	Case 17 [6]	Case 18 [8]	Case 19 [3]
Structural eye anomaly	Colobomata microphthalmos	-	-	-	-	-	-	-	-	-	-	-	-	-	-	-	-	-	-	-

Visual defect	Exotropia cataract	Myopia	-	Myopia	-	Myopia	Myopia	-	Myopia	Myopia	Myopia	Myopia		High myopia				Severe amblyopia and hyperopia		Exotropia

Dysmorphic features	-	+	+	+	+	+	+	+	+	+	+	+	+	+	+	+	+	+	+	+

Cardiac anomaly	-	+	-	-	+	+	-	+	+	-	+	+	+	+	-	+	+	-	+	-

Genital anomaly	-	+	+	-	-	-	-	-	+	-	-	-	+	-	-	-	-	+	-	+

Hypotonia	-	+	-	-	-	+	-	-	-	+	+	+	+	+	-	-	+	-	-	-

Renal anomaly	-	+	-	-	-	-	-	-	+	-	-	-	+	-	-	-	+	-	-	-

MSK anomaly	-	+	+	-	+	+	-	-	+	+	-	+	-	+	+	+	+	+	+	-

Neuro-anomaly	-	-	-	-	-	+	+	-	-	-	-	+	-	-	-	+	+	-	+	+

GIT anomaly	-	-	-	-	-	-	+	-	-	-	+	-	-	-	-	-	-	-	-	-

Hearing impairment	-	-	+	-	+	-	-	-	-	+	-	-	-	-	-	-	+	-	+	-

IUGR	-	-	+	-	-	+	-	-	+	-	-	-	-	-	-	-	-	+	-	-

Feeding issues	-	-	-	-	-	-	-	+	-	-	-	-	-	-	+	+	-	-	-	-

Developmental delay	+	-	+	+	-	-	+	-	-	+	+	+	+	+	+	+	-	+	+	+

Behavioural issues	-	-	-	-	+	-	-	-	-	-	-	-	-	-	+	-	-	-	-	+

Intellectual impairment	-	-	-	-	+	-	-	-	-	+	+	-	-	+	+	-	-	+	+	+

Deletion size and breakpoint	arr[GRCh37] 19p13.3 (4399876_ 4933228)x1	0.81 Mb Arr cgh 19p13.3 (217145– 1027368) x1.ish Del(19) (p13.3p13.3) (RP11-575H1-)pat	0.73 Mb Arr cgh 19p13.3 (217117– 950391) x1.ish Del(19) (p13.3p13.3) (RP11-575H1-)mat	0.73 Mb Arr cgh 19p13.3 (217117– 950391) x1.ish Del(19) (p13.3p13.3) (RP11-575H1-)mat	0.73 Mb Arr cgh 19p13.3 (217117– 950391) x1.ish Del(19) (p13.3p13.3) (RP11-575H1-)mat	0.86 Mb arr cgh 19p13.3 (217117– 1083955) x1.ish del(19 (p13.3p13.3) (RP11-575H1-)	0.82 Mb Arr cgh 19p13.3 (217146- 1045590) x1.ish Del(19) (p13.3p13.3) (RP11-575H1-)	0.13 Mb Arr cgh 19p13.3 (217117– 353272)x1	0.65 Mb Arr cgh 19p13.3 (736690– 1395289) x1.ish Del(19) (p13.3p13.3) (RP11-878J15-)	610 kb Arr cgh 19p13.3 (686,663– 1,297,499)	1 Mb Arr 19p13.3 (3,143,283– 4,149,860)	880 kb Arr 19p13.3 (3,256,944– 4,136,989)	792 kb Arr 19p13.3 (3,814,392– 4,605,977)	1.1 Mb Arr chr19 (213,080– 1,322,552)	1.7 MB Arr chr19 (3,870,103– 4,044,035)	1.612 Mb Arr 19p13.3 (1,890,631– 1,932,579)	1.25 Mb Arr 19p13.3 (3.325– 4.578 Mb)	0.81 Mb Arr 19p13.3 (39.27– 45.78 Mb)	5 Mb Arr chr19p13.3 (1,103,412– 1,253,309)	710 kb Arr chr (1,118,914–1,120,329) with a further centromeric deletion (1,829,934-1,837,061)
